# Editorial: Cellular and molecular mechanisms in social and repetitive behaviours: a focus on cortico-striatal circuitry

**DOI:** 10.3389/fncel.2024.1470882

**Published:** 2024-08-08

**Authors:** Ilaria Morella, Riccardo Brambilla, Yann Herault

**Affiliations:** ^1^Dipartimento di Biologia e Biotecnologie “Lazzaro Spallanzani”, Università di Pavia, Pavia, Italy; ^2^Division of Psychological Medicine and Clinical Neurosciences, School of Medicine, College of Biomedical and Life Sciences, Cardiff University, Cardiff, United Kingdom; ^3^INSERM U964 Institut de Génétique et de Biologie Moléculaire et Cellulaire (IGBMC), Illkirch-Graffenstaden, Alsace, France

**Keywords:** neurodevelopmental disorders (NDDs), repetitive/stereotyped behaviors, social behavior, cortico-striatal circuitry, copy number variants (CNV)

This Research Topic presents a collection of articles and reviews exploring the neural underpinnings of social and repetitive behaviors at the circuit, cellular, and molecular levels. Special emphasis is placed on the pathophysiological role of the basal ganglia in modulating these behaviors.

The basal ganglia, a group of subcortical nuclei including the striatum, nucleus accumbens (NAc), globus pallidus, and subthalamic nucleus, control a wide range of behavioral processes, spanning from motor behavior to instrumental learning, repetitive/stereotyped behavior, reward, and drug addiction (Cerovic et al., 2013). Recent evidence also supports the involvement of both the dorsal striatum and NAc in social behavior (Baez-Mendoza and Schultz, 2013; Dai et al., 2022; Di et al., 2022; Le Merrer et al., 2024).

Imaging studies conducted in experimental models and patients indicate significant alterations in cortico-striatal projections in autism spectrum disorder (ASD) (Di Martino et al., 2011; Fuccillo, 2016; Abbott et al., 2018). Dysregulated dopaminergic transmission underlies the “dopamine hypothesis” of schizophrenia, with the striatum playing a crucial role (McCutcheon et al., 2019). In this Research Topic, we present a recent study by Cao et al. which aims to elucidate the neural underpinnings of early-onset schizophrenia, a neurodevelopmental disorder in young children associated with more severe symptoms and poorer prognosis. The authors conducted a neuroimaging study in first-episode, medication-naïve patients, observing an abnormal enhancement of resting-state functional connectivity of the NAc with several brain regions implicated in auditory/visual processing, sensorimotor integration, and cognitive functions.

The striatum is key to repetitive/stereotyped behavior, a hallmark of neurodevelopmental disorders and other neuropsychiatric conditions (Gandhi and Lee, 2020). The review by Burton et al. provides an in-depth discussion of the striatal alterations underlying repetitive behaviors at the circuit and cellular levels, focussing on different cell types and striatal compartments (e.g., matrix vs. striosomes), as well as epigenetic factors. The authors focus on neurodevelopmental disorders (ASD and Tourette's syndrome), obsessive-compulsive disorder (OCD), as well as pre-manifest Huntington's disease, where early striatal degeneration may explain obsessive-compulsive symptoms preceding motor impairments.

Recent work by Ferhat et al. demonstrates that stereotyped behavior correlates with a size imbalance between the striosomal and matrix compartments in the striatum of an ASD mouse model (Shank3^Δ11/Δ11^). The striosomes are significantly enlarged in Shank3 mutants and overexpress glutamate decarboxylase Gad65. RNA sequencing identified several differentially expressed genes in Shank3 mutants, mainly in the striatum, further supporting the vulnerability of this brain area in ASD-related deficits.

The review by Cording and Bateup emphasizes how the striatum is involved in ASD-related stereotypies, but also in other motor abnormalities, such as changes in gait, balance, coordination and motor skills learning. This review extensively discusses the use of the accelerating rotarod task to assess motor learning and coordination in mouse models with ASD-linked mutations. Interestingly, although rotarod performance varies, some models exhibit improved motor learning and coordination, potentially due to an increased cortico-striatal drive.

The role of different striatal cell populations in ASD-related behaviors is particularly relevant but still poorly understood. Medium spiny neurons (MSNs), which constitute more than 90% of the striatal neuronal population, include D1- and D2-expressing MSNs. These two populations form the direct and indirect pathways, respectively (Lanciego et al., 2012). As discussed by Soghomonian, substantial evidence in experimental models supports the distinct roles of D1 and D2 MSNs in social and repetitive behaviors. An imbalance between these pathways may thus contribute to ASD-related social deficits and stereotyped behavior.

Recent research by Giua et al. has identified cell-specific effects in MSNs subtypes, in a mouse model of Fragile X Syndrome (FXS), a common monogenic cause of autism and inherited intellectual disability. Specifically, D1 and D2 MSNs in the NAc core of FXS mice exhibited significant alterations in membrane properties and action potential kinetics. These electrophysiological changes disrupt the typical functional separation between D1 and D2 MSNs, potentially contributing to FXS-associated pathological features.

Further research underscores the involvement of dopaminergic signaling in neurodevelopmental disorders. The CHL1 gene, located at 3p26.1 and part of the immunoglobulin family, is linked to these disorders and has been identified as an interactor of D2 receptors, reducing the internalization of the short D2 receptor isoform (Kotarska et al., 2020). The study carried out by Fernandes et al. in CHL1-deficient mice highlighted the role of CHL1 in regulating various D2-dependent behaviors, with effects observed in both sex-dependent and sex-independent manners.

Copy number variants (CNVs) at the 16p11.2 chromosomal region are implicated in neurodevelopmental disorders, intellectual disability, ASD, and epilepsy (Rein and Yan, 2020). Leone et al. provide an up-to-date overview of these conditions, discussing findings from human studies, animal models, and cellular models. Converging evidence supports the role of cortico-striatal circuitry in the pathophysiology of 16p11.2 CNVs, with a potential vulnerability observed in males, as evidenced by mouse studies. This review also provides a detailed description of the genes within the 16p11.2 region, including non-coding RNAs. Additionally, pharmacological approaches targeting these genes, as well as interventions beyond the chromosomal locus, are discussed.

In the context of 16p11.2 deletion, Rusu et al. emphasize the importance of monitoring mouse behavior over extended periods to provide a more refined analysis of behavioral deficits and better mimic everyday-life deficits in patients. By monitoring spontaneous social interactions over 2–3 days, the authors found that the social domain was differentially affected between sexes depending on the social context. For instance, 16p11.2 deletion males were more impacted than females in the social domain when tested in quartets of familiar individuals, whereas 16p11.2 deletion females displayed significant alterations in social behaviors when tested in pairs of familiar individuals.

Altogether, this Research Topic highlights the crucial role played by the basal ganglia and related neural circuits in social and repetitive behaviors, providing insights into the pathophysiology of various neurodevelopmental and neuropsychiatric conditions. Understanding the neural mechanisms underpinning social and motor behaviors will be crucial to develop more effective interventions for these conditions.

## Author contributions

IM: Writing – original draft, Writing – review & editing. RB: Writing – original draft, Writing – review & editing. YH: Writing – original draft, Writing – review & editing.
